# Unexpected Enhancement of Cytotoxicity of Cisplatin in a Rat Kidney Proximal Tubular Cell Line Overexpressing Mitochondrial Glutathione Transport Activity

**DOI:** 10.3390/ijms23041993

**Published:** 2022-02-11

**Authors:** Lawrence H. Lash

**Affiliations:** Department of Pharmacology, Wayne State University School of Medicine, Detroit, MI 48201, USA; l.h.lash@wayne.edu; Tel.: +1-313-577-0475

**Keywords:** glutathione, transport, kidney, mitochondria, cisplatin, cytotoxicity, 2-oxoglutarate carrier, oxidative stress

## Abstract

In previous studies, we identified the two principal transporters that mediate the uptake of glutathione (GSH) from cytoplasm into the mitochondrial matrix of rat kidney proximal tubular cells. We hypothesized that genetic modulation of transporter expression could markedly alter susceptibility of renal proximal tubular cells to a broad array of oxidants and mitochondrial toxicants. Indeed, we previously showed that overexpression of either of these transporters resulted in diminished susceptibility to several chemicals. In the present work, we investigated the influence of overexpression of the mitochondrial 2-oxoglutarate carrier (OGC) in NRK-52E cells on the cytotoxicity of the antineoplastic drug cisplatin. In contrast to previous results showing that overexpression of the mitochondrial OGC provided substantial protection of NRK-52E cells from injury due to several toxicants, we found a remarkable enhancement of cellular injury from exposure to cisplatin as compared to wild-type NRK-52E cells. Despite the oxidative stress that cisplatin is known to cause in the renal proximal tubule, the increased concentrations of mitochondrial GSH associated with OGC overexpression likely resulted in increased delivery of cisplatin to molecular targets and increased cellular injury rather than the typical protection observed in the previous work.

## 1. Introduction

Cisplatin (also known as cis-diamminedichloroplatinum (II) or CDDP) is a widely used anticancer drug that was approved for use in treating several cancers by the U.S. Food and Drug Administration in 1978. CDDP is also considered by the World Health Organization as one of the 100 essential drugs [1]. Despite the continued utility of CDDP in chemotherapy [2,3], two major problems in its use are tumor resistance and off-target side effects [4]. Among the off-target side effects, kidney injury is one of the most prominent [5,6,7,8,9] and has been extensively studied both in terms of developing biomarkers to detect such injury during CDDP therapy and to design approaches to protect against it so that therapy can continue.

Several factors and cellular mechanisms have been identified as critical determinants of CDDP-induced nephrotoxicity. These have included active uptake by proximal tubular cells by the organic cation transporter 2 (OCT2; Slc22a2) and the copper transporter 1 (CTR1; Slc31a1) [10,11], generation of oxidants and production of a cellular state of oxidative stress [12,13,14,15,16,17,18,19,20,21,22,23,24], endoplasmic reticulum stress [25,26], inflammation [27,28], various processes in mitochondria that can lead to energetic failure [28,29,30,31,32,33,34,35,36,37,38,39,40], and cell death involving autophagy and other pathways [34,41,42,43,44,45,46]. This list of cellular processes and functions that are adversely impacted by CDDP exposure does not, however, explain the molecular mechanism by which CDDP causes renal cellular injury. As noted by George et al. [7], once inside the proximal tubular cell, the chloride atoms of CDDP become labile and are replaced by water molecules to become hydrated, electrophilic species that can then target cellular macromolecules. One of the intracellular targets for this hydrated, electrophilic form of CDDP appears to be glutathione (GSH), as conjugation of CDDP with GSH has been reported in rodent kidney studies [47,48,49].

Although conjugation with GSH is cytoprotective for many reactive electrophiles, Hanigan and colleagues [48,49,50,51,52,53] and others [54] have provided data in several in vivo and in vitro studies that led them to conclude that the CDDP-GSH conjugate follows a bioactivation mechanism in the renal proximal tubule that has been well-established for halogenated solvents such as tri- and perchloroethylene [55]. The key data supporting this include prevention of nephrotoxicity in vivo and cytotoxicity in vitro by inhibition of γ-glutamyltransferase (GGT) or cysteine conjugate β-lyase (CCBL) activities. In some contrast with these studies, Wainford et al. [56] examined CDDP nephrotoxicity in Sprague-Dawley rats and cytotoxicity in isolated proximal tubular cells from rats and humans. While inhibition of GGT activity protected rats in vivo from CDDP-induced nephrotoxicity, inhibition of any of the subsequent enzymes in the pathway, including CCBL activity, had no impact on CDDP-induced nephrotoxicity in vivo or CDDP-induced cytotoxicity in vitro. Accordingly, these authors concluded that while GGT is a key enzyme involved in mediating CDDP-induced nephrotoxicity, the CDDP-GSH conjugate does not follow the bioactivation mechanism established for halogenated solvents. Based on the above discussion, the potential protective or bioactivation role of GSH in CDDP-induced nephrotoxicity remains unclear and is likely more complex than simply an antioxidant effect of GSH or as a metabolic intermediate.

Despite the precise role of GSH in CDDP-induced nephrotoxicity being unclear, it is well-established that both redox status—GSH status in particular—and mitochondrial function in renal proximal tubular cells are key components in the renal response to CDDP exposure [12,13,14,15,16,17,18,19,20,21,22,23,24,28,29,30,31,32,33,34,35,36,37,38,39,40]. To attempt to address the role of GSH and renal mitochondria in the disposition and mechanism of action of CDDP in renal proximal tubular cells, we investigated the impact of altered mitochondrial GSH status on CDDP-induced cytotoxicity. The content of renal mitochondrial GSH is determined exclusively by transport of GSH from the cytoplasm into the mitochondrial matrix and is mediated by two organic anion carriers on the mitochondrial inner membrane, the dicarboxylate carrier (DCC; Slc25a10), and the 2-oxoglutarate carrier (OGC; Slc25a11) [57]. In previous work, we demonstrated that transient overexpression of the DCC in a proximal tubular cell line, Normal Rat Kidney-52 Epithelial (NRK-52E) cells, derived from rat kidney, increased mitochondrial GSH content and protected those cells from injury due the oxidant *tert*-butyl hydroperoxide (tBH) or the nephrotoxic metabolite of trichloroethylene, *S*-(1,2-dichlorovinyl)-L-cysteine (DCVC) [58]. Similarly, stable transfection of NRK-52E cells to overexpress the OGC resulted in marked enhancement of mitochondrial transport and accumulation of GSH both in mitochondria and in the cell as a whole and protection from apoptosis induced by either tBH or DCVC [59]. In contrast, stable overexpression in NRK-52E cells of a double-cysteine mutant of the OGC (rOGC-C221,224S) with markedly reduced mitochondrial GSH transport function did not protect the cells from injury. Instead, those cells exhibited enhanced susceptibility to injury as compared to the wild-type cells [59].

In the present work, we hypothesized that NRK-52E cells that stably express high levels of the OGC should be protected from cellular injury due to CDDP exposure. Responses of both wild-type (NRK-52E-WT) and OGC-overexpressing cells (NRK-52E-OGC) to CDDP were assessed by measurements of cellular necrosis, apoptosis, and total cellular concentrations of GSH and cellular morphology. Rather than demonstrate the expected protection by OGC overexpression, enhanced cytotoxicity from CDDP exposure was observed as compared to WT cells.

## 2. Results

### 2.1. CDDP-Induced Necrotic Cell Death

**Figure 1:**
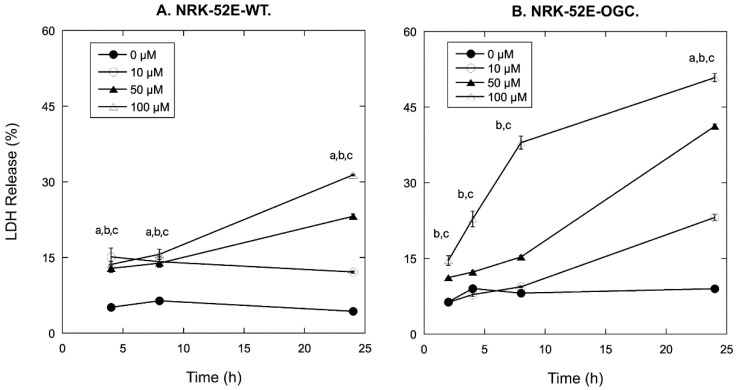
Time and concentration dependence of CDDP-induced LDH release. Confluent, wild-type NRK-52E (NRK-52E-WT) (**A**) and confluent, NRK-52E cells overexpressing the OGC carrier (NRK-52E-OGC) (**B**) were incubated in 24-well cell culture plates for up to 24 h with 0, 10, 50, or 100 µM CDDP. LDH activity was measured in both culture medium and total cells. Values are means ± SEM of percent LDH release for 4 individual incubations for controls (0 µM) and 3 individual incubations for cells incubated with 10, 50, or 100 µM CDDP. a,b,c = Significantly different (*p* < 0.05) from 0-µM control for 10, 50, and 100 µM CDDP, respectively, at the same time point.

Cell death by necrosis was assessed by measurement of release of lactate dehydrogenase (LDH). As shown in Figure 1A, NRK-52E-WT cells incubated for 4, 8, or 24 h with 0, 10, 50, or 100 µM CDDP exhibited modest amounts of LDH release at 4 and 8 h but then exhibited significant increases in LDH release with increasing concentrations of CDDP at 24 h. In contrast with expectations that NRK-52E-OGC cells would be resistant to CDDP-induced necrosis, these cells exhibited markedly higher amounts of LDH release as compared to those measured in the NRK-52E-WT cells (Figure 1B). Whereas maximal fractions of LDH release in NRK-52E-WT cells incubated with 10, 50, or 100 µM CDDP for 24 h were 12.1, 23.2, and 31.4%, respectively, maximal fractions of LDH release in NRK-52E-OGC cells similarly incubated were 23.1, 41.2, and 50.8%, respectively.

### 2.2. CDDP-Induced Apoptosis

**Figure 2:**
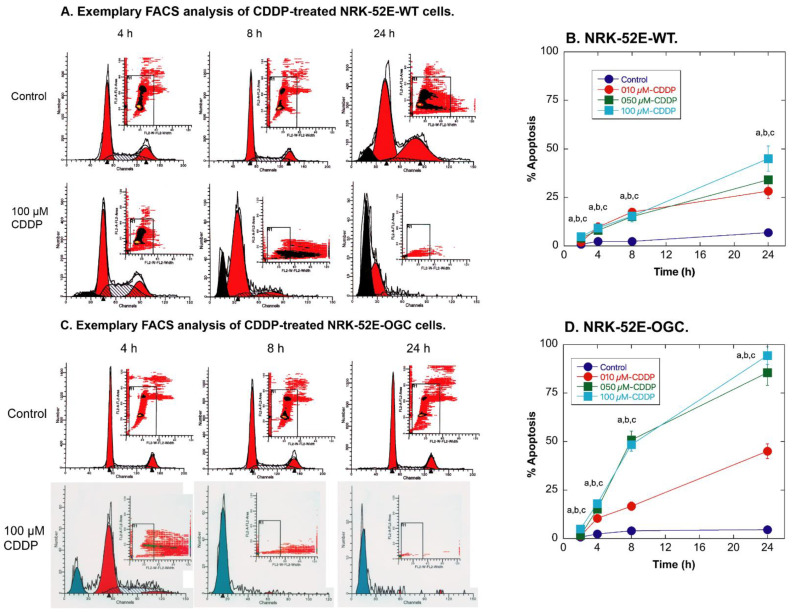
Time and concentration dependence of CDDP-induced cell cycle changes. Confluent, wild-type NRK-52E cells (NRK-52E-WT) and confluent, NRK-52E cells stably overexpressing OGC (NRK-52E-OGC) were preincubated for 24 h on 35-mm dishes with media containing 1 mM GSH and were then incubated for up to 24 h with either media (=0 µM) containing 20 µM GSH or media containing 10, 50, or 100 µM CDDP in the presence of 20 µM GSH. Cells were harvested by trypsin/EDTA treatment, washed in sterile PBS, fixed overnight in ethanol, and then stained with propidium iodide and analyzed by flow cytometry with a Becton Dickinson FACS*Calibur* flow cytometer. (**A**) Exemplary FACS analysis of NRK-52E-WT cells treated with 100 µM CDDP. (**B**) Time- and concentration-dependent apoptosis induced by CDDP in NRK-52E-WT cells. (**C**) Exemplary FACS analysis of NRK-52E-OGC cells treated with 100 µM CDDP. (**D**) Time- and concentration-dependent apoptosis induced by CDDP in NRK-52E-OGC cells. For panels B and D, a,b,c = Significantly different (*p* < 0.05) from 0-µM control for 10, 50, and 100 µM CDDP, respectively, at the same time point.

Susceptibility of NRK-52E cells to CDDP-induced cytotoxicity was further studied by assessing cell cycle status by flow cytometry (Figure 2). As with LDH release, NRK-52E-OGC cells exhibited a significantly higher amount of apoptosis than NRK-52E-WT cells incubated with the same concentrations of CDDP. Although the extent of apoptosis was higher at all concentrations of CDDP, the exemplary plots shown for cells incubated with 100 µM CDDP (Figure 2A,C) show the greater degree of cell death most clearly. Whereas NRK-52E-WT cells incubated with 100 µM CDDP for 8 h or 24 h still exhibited a large proportion of cells in the G_0_/G_1_ and S phases of the cell cycle, the NRK-52E-OGC cells similarly incubated with CDDP exhibited almost no detectable cells in the G_0_/G_1_ and S phases of the cell cycle with virtually all cells being sub-G_1_, meaning that they are almost all undergoing apoptosis. Indeed, as shown in Figure 2B, the fractions of NRK-52E-WT cells incubated for 24 h with 10, 50, or 100 µM CDDP undergoing apoptosis were 9.3, 15.4, and 45.0%, respectively. In contrast, as shown in Figure 2D, the fractions of NRK-52E-OGC cells similarly incubated with CDDP that were undergoing apoptosis were 18.1, 48.5, and 94.3%, respectively. Of note, the total amount of cell death as indicated by LDH release and apoptosis in NRK-52E-OGC cells incubated with 50 or 100 µM CDDP are >100%, suggesting that a proportion of the cells detected by propidium iodide staining and flow cytometry and designated as sub-G_1_ are likely necrotic.

### 2.3. CDDP-Induced Changes in Cellular Morphology

**Figure 3:**
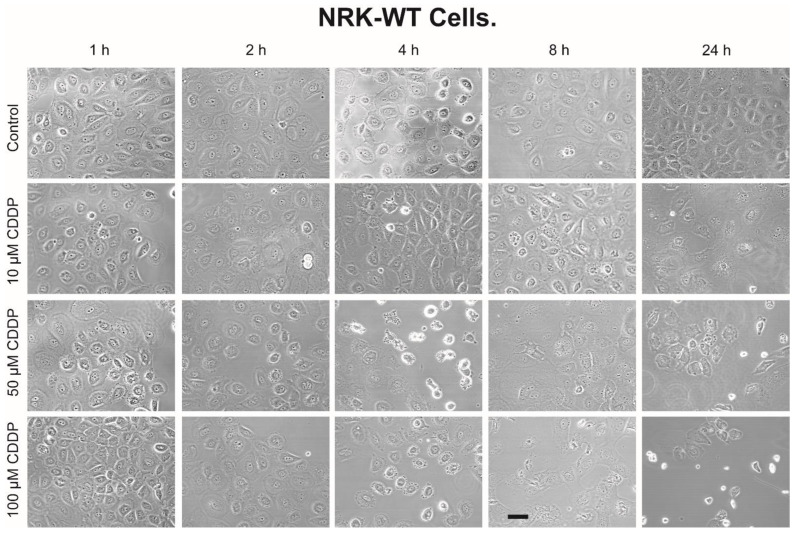
Morphology of NRK-52E-WT cells incubated with CDDP. Confluent, wild-type NRK-52E cells (NRK-52E-WT) were incubated for up to 24 h with 0, 10, 50, or 100 µM CDDP. Photomicrographs were obtained on a Zeiss Triple Laser-Scanning confocal microscope at an initial magnification of 196×. Bar = 5 µm.

**Figure 4:**
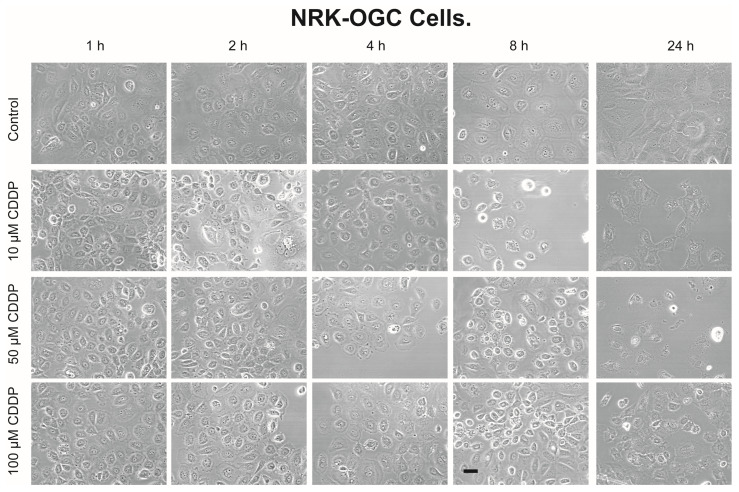
Morphology of NRK-52E-OGC cells incubated with CDDP. Confluent, NRK-52E cells overexpressing OGC (NRK-52E-OGC) were incubated for up to 24 h with 0, 10, 50, or 100 µM CDDP. Photomicrographs were obtained on a Zeiss Triple Laser Scanning confocal microscope at an initial magnification of 196×. Bar = 5 µm.

Morphology of NRK-52E-WT cells (Figure 3) and NRK-52E-OGC cells (Figure 4) incubated for up to 24 h with 0, 10, 50, or 100 µM CDDP shows the time- and concentration-dependent effects of CDDP on cellular structure and density. Differences between WT and OGC cells are not apparent at the earlier time points but are more evident at the 8- and 24-h time points. At these time points, a higher proportion of NRK-52E-OGC cells incubated with CDDP appeared to be rounded and raised from the culture surface as compared to NRK-52E-WT cells similarly incubated with CDDP or there was a more obvious formation of pyknotic or oddly shaped cells.

### 2.4. Effects of CDDP on Total Cellular GSH Concentrations

**Figure 5:**
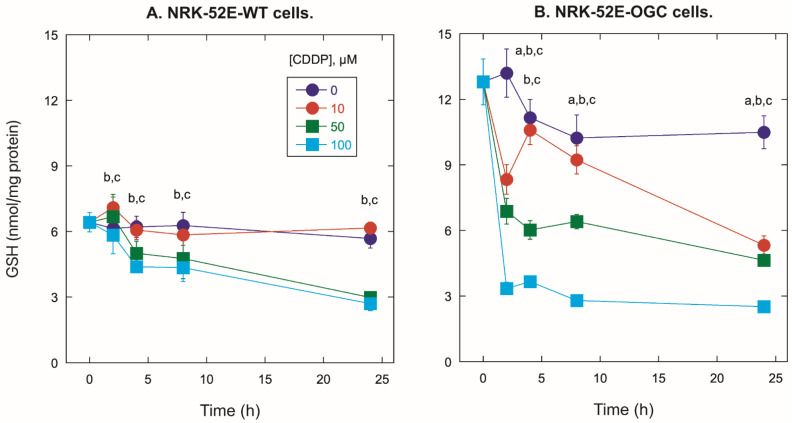
Time and concentration dependence of effects of CDDP on total cellular GSH concentration. Concentrations of total cellular GSH were determined in cells grown on collagen-coated T-25 flasks. After detachment at the indicated times by brief treatment with trypsin/EDTA, GSH content was determined by ion-exchange high-pressure liquid chromatography after derivatization with iodoacetic acid and 1-fluoro-2,4-dinitrobenzene. Results are means ± SEM of measurements from 3 separate cell cultures for NRK-52E-WT cells (**A**) and NRK-52E-OGC cells (**B**). a,b,c = Significantly different (*p* < 0.05) from 0-µM control for 10, 50, and 100 µM CDDP, respectively, at the same time point.

Assessment of total cellular GSH concentrations (Figure 5) confirms that NRK-52E-OGC cells contain approximately double the concentration of GSH as NRK-52E-WT cells. The interesting finding was that despite the markedly higher concentrations of GSH in the NRK-52E-OGC cells as compared to those in the NRK-52E-WT cells at time 0 (12.8 ± 1.1 vs. 6.42 ± 0.44 nmol/mg protein, respectively), the fraction of the decrease in GSH concentration with increasing CDDP incubation concentration was much greater in those cells as compared to the NRK-52E-WT cells (decreased by 80.4% in NRK-52E-OGC cells incubated for 24 h with 100 µM CDP vs. decreased by 57.9% in NRK-52E-WT cells similarly incubated).

## 3. Discussion

The current study focused on two factors in the interaction between CDDP and the renal proximal tubular cell, namely, mitochondria and GSH status. Previous work of ours identified the two anion carriers, the DCC and OGC, that are responsible for the critical mitochondrial pool of GSH [57]. As redox regulation [12,13,14,15,16,17,18,19,21,22,23,24,34,35,36,37,38,39,40] and mitochondrial function [4,5,6,12,29,30,33,40,47] are critically involved in the mechanism of proximal tubular cytotoxicity caused by CDDP, we hypothesized that increasing the mitochondrial and cellular concentrations of GSH could be protective. We had previously shown that overexpression of either the DCC [58] or OGC [59] in a rat proximal tubular cell line, NRK-52E cells, significantly protected these cells from injury due to an oxidant, tBH, and a mitochondrial toxicant, DCVC, that acts by both oxidative stress and formation of a reactive electrophile. Although NRK-52E cells exhibit many properties of the in vivo rat proximal tubule, these cells exhibit relatively low expression and activity of mitochondrial and plasma membrane GSH transporters [60]. This limitation in the WT cell line is actually advantageous as it provides a low baseline against which cells that overexpress any of these transporters can be compared. Another study of ours [61] in primary cultures of rat proximal tubular cells derived from the remnant kidneys of uninephrectomized rats showed that overexpression of the DCC or OGC reverted these cells from a hypertrophied, pro-oxidant state to more normally functioning proximal tubular cells with a diminished state of oxidative stress.

CDDP causes proximal tubular cell death by multiple pathways, including autophagy, apoptosis, and necrosis [34,41,42,43,44,45,46]. We assessed the ability of a range of concentrations of CDDP to cause proximal tubular cell death by either necrosis (LDH release; Figure 1) or apoptosis (cell cycle analysis by propidium iodide staining and flow cytometry; Figure 2). Our rationale for choosing the concentrations to which the NRK-52E cells were exposed was three-fold. First, plasma levels of CDDP in patients on a chemotherapy regimen with CDDP have been reported to be between 1.9 and 11 µM [62,63,64,65,66]. Second, studies of ours in primary cultures of human proximal tubular cells found that a concentration of 20 µM CDDP exhibited little or no cytotoxicity whereas a concentration of 90 µM CDDP was moderately cytotoxic [66]. Finally, mammalian kidneys can accumulate CDDP to concentrations as much as fivefold higher than those in plasma [5,6,28]. Accordingly, we incubated the two types of NRK-52E cells with 10, 50, or 100 µM CDDP to obtain a range of cytotoxicity from low to moderate. NRK-52E-WT cells exhibited minimal increases in LDH release (<15%) through 24 h at 10 µM, and moderate increases in LDH release of approximately 20% and 30% at 24 h with 50 µM and 100 µM CDDP, respectively. In contrast, the NRK-52E-OGC cells exhibited moderate LDH release (~20%) at 24 h with 10 µM CDDP, moderate LDH release (~20%) at 4 h with 100 µM CDDP, and high amounts of LDH release (>40%) at 8 h and 24 h with 100 µM CDDP and 24 h with 50 µM CDDP.

A similar disparity in the susceptibility of the two NRK-52E cell lines was observed with cell cycle analysis. Whereas all three concentrations of CDDP produced moderate increases in the proportion of sub-G_1_ cells after 8 h (15–20%) and 24 h (25–45%) in the WT cells, CDDP produced much greater increases in sub-G_1_ cells at all time points from 4 h to 24 h. Both 50 and 100 µM CDDP caused more than 80% of the cells to undergo apoptosis by 24 h in the OGC-overexpressing cells. As noted above, some of the cells identified as sub-G_1_ are possibly necrotic as the total fraction of cells indicated as “dead” (i.e., necrotic + apoptotic) was >100%. Analysis of cellular morphology (Figure 3 and Figure 4) also suggested a higher degree of cell damage in NRK-52E-OGC cells, especially at the later time points.

The key question that arises from these data is: What is the mechanism underlying the greater susceptibility of OGC-overexpressing cells to CDDP? The obvious difference between the two cell lines is their mitochondrial and cellular GSH status. Stable overexpression of the OGC in NRK-52E cells resulted in approximately a 75% and 400% increase in the initial rate of mitochondrial GSH uptake with 5 mM and 10 mM GSH, as compared to WT cells [59]. In the present study, total cellular concentrations of GSH were measured over a period of 24 h in cells incubated with 0, 10, 50, or 100 µM CDDP (Figure 5). Although the direct effect of OGC overexpression is expected to be an increase in mitochondrial GSH concentrations, NRK-52E-OGC cells exhibited approximately two-fold higher total cellular GSH concentrations than NRK-52E-WT cells. A likely explanation for the increase in total cellular GSH concentration is an upregulation of the glutamate-cysteine ligase, which is the rate-limiting enzyme responsible for the initial step in GSH synthesis in the cytoplasm [67]. With stable mitochondrial OGC overexpression, large amounts of cytoplasmic GSH are transported into mitochondria, resulting in lower concentrations of GSH in the cytoplasm. With the feedback inhibition of the glutamate-cysteine ligase diminished due to the marked decrease in cytoplasmic GSH, an increase in expression and activity could occur, thereby resulting in a significant increase in cytoplasmic and hence total cellular GSH concentrations.

Despite the higher total cellular concentrations of GSH in the NRK-52E-OGC cells, the fractions by which these concentrations decreased with increasing incubation time and increasing CDDP concentration were greater than those in the NRK-52E-WT cells. Thus, the higher total cellular, and presumably mitochondrial, GSH concentrations were not protective against CDDP-induced cytotoxicity. Moreover, despite the two cell types having similar concentrations of GSH at later incubation times, the NRK-52E-OGC cells exhibited a higher amount of necrotic and apoptotic cells than the NRK-52E-WT cells. This suggests that overexpression of the mitochondrial OGC protein may result in increased susceptibility to CDDP by a process other than just the mitochondrial or cellular GSH concentrations.

A recent review on the mechanisms of CDDP resistance in cancer cells by Devarajan et al. [68] highlighted one mechanism by which cancer cell resistance develops that involves increased formation of reactive oxygen species, subsequent increased expression of the transcription factor nuclear factor-erythroid factor 2-related factor 2 (Nrf2), and subsequent induction of the two subunits of γ-glutamyl-cysteine synthetase, which in turn results in increased concentrations of GSH. In cancer cells, Devarajan et al. [68] concluded that this ultimately results in increased detoxification of CDDP. It would appear that in renal proximal tubular cells, however, the ultimate result of increased cellular concentrations of GSH is not increased detoxification, but rather, increased bioactivation. The markedly higher concentrations of GSH in the OGC-overexpressing cells may also result in increased accumulation of CDDP, which is something that needs to be investigated further. This finding would seem to be consistent with the work of Hannigan and colleagues [48,49,50,51,52,53] regarding the role of GSH conjugation in renal bioactivation of CDDP. Although the role of the CCBL in this bioactivation process has been questioned [56], the protective effect of inhibition of the GGT-dependent metabolism of the CDDP-GSH conjugate has been confirmed in multiple studies by multiple groups. This fact is, therefore, consistent with GSH conjugation of CDDP playing a role in its bioactivation rather than its detoxification.

## 4. Materials and Methods

### 4.1. Materials

Restriction enzymes for PCR, other enzymes (e.g., DNA polymerases and T7 RNA polymerase), and plasmids were purchased from New England Biolabs (Beverly, MA, USA), Promega (Madison, WI, USA), and Gibco-BRL/Life Technologies (Gaithersburg, MD, USA). PCR primers were custom synthesized by Integrated DNA Technology, Inc. (Coralville, IA, USA). Cloning vectors (pGEM-T Easy and pRESET, pcDNA3.1/V5-His-TOPO) were purchased from Promega and Invitrogen (Carlsbad, CA, USA). Materials for gel electrophoresis (SDS, acrylamide, and agarose buffers) were purchased from Bio-Rad (Hercules, CA, USA) or Sigma-Aldrich (Milwaukee, WI, USA). ProBond nickel-chelating resin was purchased from Invitrogen. NRK-52E cells (catalog no. CRL-1571) and cell culture medium (catalog no. 30-2002; Dulbecco’s modified Eagle’s medium with 4 mM L-glutamine adjusted to contain 1.5 g/L sodium bicarbonate, 4.5 g/L glucose, and 1.0 mM sodium pyruvate), and 10% (*w*/*v*) bovine calf serum were purchased from the American Type Culture Collection (Manassas, VA, USA). Antibodies to the His_6_-fusion protein was purchased from Invitrogen or QIAGEN (Valencia, CA, USA). Double-distilled, deionized water was used for all experiments. CDDP was purchased from Sigma-Aldrich (catalog no. P4394). Stock solutions were soluble in aqueous solutions with application of heat. All other chemicals and reagents were purchased from commercial vendors and were of the highest purity available.

### 4.2. Amplification of Rat Kidney Mitochondrial OGC cDNA by RT-PCR

Rat kidney mitochondrial OGC cDNA was amplified and expressed in *E. coli* as previously described [59]. A brief overview of the procedure is presented here. Total rat kidney RNA was reverse transcribed and amplified with forward and reverse primers based on the complete cDNA sequence (1149 bp) for the heart mitochondrial OGC protein from the Norway rat (GenBank accession no. NM_022398). RT-PCR was conducted, the product was loaded onto an agarose gel, the gel was stained with ethidium bromide, and the bands were visualized under UV light. The 1149-bp product was ligated into a T-A cloning vector (pGEM-T Easy) for transformation. The sequence of the PCR product was confirmed by automated DNA sequencing. The full-length cDNA for rat kidney OGC was subcloned into the pRSET T7 expression vector for high-level expression in *E. coli* as a His_6_-fusion protein. The OGC-His_6_ fusion protein was obtained from the inclusion body fraction and was further purified by passage over a nickel-chelating resin.

### 4.3. Culture and Transfection of NRK-52E Cells

NRK-52E-WT and NRK-52E-OGC cells were cultured on collagen-coated polystyrene T-25 culture flasks, 35-mm dishes, or in 24-well plates, depending on the experiment, with Dulbecco’s modified Eagle’s medium containing 4% (*w*/*v*) L-glutamine, 1.5 g/L sodium bicarbonate, 4.5 g/L glucose, 1 mM sodium pyruvate, and 10% (*v*/*v*) bovine calf serum in an atmosphere of 5% CO_2_, 95% air at 37˚C. On reaching confluence (5–9 days), subcultures were prepared by a 15-min treatment with 0.02% EDTA, 0.05% trypsin, and cells were replated at a density of 4 × 10^4^ cells/cm^2^. NRK-52E cells overexpressing the rat OGC protein were generated as previously described [59]. Briefly, plasmid DNA was purified and the cDNA was subcloned into the pcDNA3.1/V5-His-TOPO vector. Stable transfection was achieved with FuGENE 6 from Roche Applied Science (Indianapolis, IN, USA). Stable transfectants were selected with Geneticin (G418) and protein expression was verified with the antibody to His_6_-fusion proteins and Western blot analysis.

### 4.4. Assay of Cellular Necrosis by LDH Release

Cells were incubated in 24-well plates for up to 24 h. At the indicated times, aliquots from extracellular media and those from cells solubilized with Triton X-100 were obtained for measurement of LDH activity. Activities were measured spectrophotometrically at 340 nm as NADH oxidation after addition of pyruvate and NADH. Percentage of LDH release was calculated by the formula:

% LDH release = (LDH activity in media/(LDH activity in media + LDH activity in cells)) × 100%(1)

### 4.5. Flow Cytometry Analysis of Cell Cycle

Both WT and OGC NRK-52E cells were grown on 35-mm culture dishes until approximately 80–90% confluence. Cells were pretreated for 24 h with 1 mM GSH and were then treated for up to 24 h with either media (=0 µM or control) or various concentrations of CDDP in the presence of 20 µM GSH. Procedures for preparation of samples for analysis of cell-cycle status by flow cytometry were as previously described [58,59]. After harvesting, fixation in ethanol, and staining with propidium iodide, samples were analyzed by flow cytometry using a Becton Dickinson FACS*Calibur* flow cytometer (Becton Dickinson, San Jose, CA, USA). Analyses were performed with 20,000 events per sample using the ModFit LT version 2 for Macintosh data acquisition software package (Verity Software; distributed by Becton Dickinson, San Jose, CA, USA). Fractions of apoptotic cells were determined by analysis of sub-G_1_ (subdiploid) peaks whereas other phases of the cell cycle (G_0_/G_1_, S, and G_2_/M) were also calculated.

### 4.6. Confocal Microscopy

To obtain photomicrographs of NRK-52E cells treated with various concentrations of CDDP, cells were grown on collagen-coated, 35-mm culture dishes and were viewed with a Zeiss Triple-Laser Scanning confocal microscope (LSM30) (Carl Zeiss AG, Jena, Germany). Initial magnification was 196×.

### 4.7. Measurement of Total Cellular GSH Content by HPLC

Cellular content of GSH in cells grown on T-25 flasks was determined by ion-exchange high-pressure liquid chromatography using a Waters µBondapak amine column (Waters Corporation, Milford, MA, USA) after derivatization of samples with iodoacetic acid and 1-fluoro-2,4-dinitrobenzene according to previously described methods [69,70]. Derivatives were detected by absorbance at 365 nm and were compared to authentic standards. Assay limit of detection was 50 pmol.

### 4.8. Data Analysis

Results are expressed as means ± SEM of measurements from the indicated number of cell preparations. Statistical analysis was done using GraphPad Prism software version 9 (GraphPad Software, San Diego, CA, USA). Significant differences between mean values of controls and treated samples were first assessed by a one-way or two-way analysis of variance. When significant *F* values were obtained with the analysis of variance, the Fisher’s protected least-significance *t*-test was performed to determine which means were significantly different from one another, with two-tail probabilities < 0.05 considered significant.

## 5. Conclusions

The findings from the present work illustrate the principle that approaches or mechanisms that are typically thought of as being cytoprotective may actually have the opposite effect in specific cases. With CDDP, part of the rationale to hypothesize that enhancement of the cellular concentrations of a major thiol antioxidant would be protective is based on the extensive data showing that CDDP generates reactive oxygen species, produces oxidative stress, and causes disturbances in renal mitochondrial function. The unique aspects of CDDP handling and metabolism by the kidneys, however, likely account for the finding that significant enhancement of mitochondrial and total cellular GSH enhance CDDP-induced cytotoxicity. These unique aspects likely include formation of a CDDP-GSH conjugate, and its transport and further metabolism by renal enzymes of the classic mercapturic acid pathway. Additional measurements of CDDP metabolites and cellular or mitochondrial accumulation of either CDDP or Pt may provide some further insight into the nephrotoxicity of CDDP. The importance of this lies in the continued clinical use of CDDP in cancer chemotherapy and the dose limitations caused by CDDP side effects, of which nephrotoxicity is prominent.

In summary, the present work clearly shows the following: (i) Stable overexpression of the OGC results in significantly greater amounts of time- and concentration-dependent necrotic cell injury due to CDDP, as shown by LDH release (Figure 1). (ii) Stable overexpression of the OGC results in significantly greater amounts of time- and concentration-dependent apoptosis due to CDDP, as shown by propidium iodide staining and flow cytometry (Figure 2). (iii) NRK-52E cells that stably overexpress the OGC exhibited a modestly greater extent of morphological damage from CDDP (Figure 3 and Figure 4). (iv) Despite higher total cellular concentrations of GSH than NRK-52E-wild-type cells, NRK-52E cells overexpressing the OGC exhibited more pronounced decreases in these concentrations of GSH after exposure to CDDP. (v) The greater amount of cell death exhibited by NRK-52E-OGC cells vs. NRK-52E-WT cells occurred with the same concentrations of GSH, suggesting that other processes besides just mitochondrial GSH transport were responsible.

## References

[1] World Health Organization (2021). World Health Organization Model List of Essential Medicines—22nd List.

[2] Rottenberg S., Disler C., Perego P. (2021). The rediscovery of platinum-based cancer chemotherapy. Nat. Rev. Cancer.

[3] Jin S., Guo Y., Guo Z., Wang X. (2021). Monofunctional platinum(II) anticancer agents. Pharmaceuticals.

[4] Tchounwou P.B., Dasari S., Noubissi F.K., Ray P., Kumar S. (2021). Advances in our understanding of the molecular mechanisms of action of cisplatin in cancer therapy. J. Exp. Pharmacol..

[5] dos Santos N.A.G., Rodrigues M.A.C., Martins N.M., dos Santos A.C. (2012). Cisplatin-induced nephrotoxicity and targets of nephroprotection: An update. Archiv. Toxicol..

[6] Zhang J., Ye J.-w., Tew K.D., Townsend D.M. (2021). Cisplatin chemotherapy and renal function. Adv. Cancer Res..

[7] George B., Joy M.S., Aleksunes L.M. (2018). Urinary protein biomarkers of kidney injury in patients receiving cisplatin chemotherapy. Exp. Biol. Med..

[8] Kim H.R., Park J.H., Lee S.H., Kwack S.J., Lee J., Kim S., Yoon S., Kim K.-B., Lee B.M., Kacew S. (2022). Using intracellular metabolic profiling to identify novel biomarkers of cisplatin-induced acute kidney injury in NRK-52E cells. J. Toxicol. Environ. Health A.

[9] Perse M., Veceric-Haler Z. (2018). Cisplatin-induced rodent model of kidney injury: Characteristics and challenges. BioMed Res. Int..

[10] Aleksunes L.M., Augustine L.M., Scheffer G.L., Cherrington N.J., Manautou J.E. (2008). Renal xenobiotic transporters are differentially expressed in mice following cisplatin treatment. Toxicology.

[11] Ciarimboli G., Ludwig T., Lang D., Pavenstädt H., Koepsell H., Piechota H.-J., Haier J., Jaehde U., Zisowsky J., Schlatter E. (2005). Cisplatin nephrotoxicity is critically mediated via the human organic cation transporter 2. Am. J. Pathol..

[12] Baliga R., Zhang Z., Baliga M., Ueda N., Shah S.V. (1998). In vitro and in vivo evidence suggesting a role for iron in cisplatin-induced nephrotoxicity. Kidney Int..

[13] Dickey D.T., Wu Y.J., Muldoon L.L., Neuwelt E.A. (2005). Protection against cisplatin-induced toxicities by N-acetylcysteine and sodium thiosulfate as assessed at the molecular, cellular, and in vivo levels. J. Pharmacol. Exp. Ther..

[14] Hajian S., Rafieian-Kopaei M., Nasri H. (2014). Renoprotective effects of antioxidants against cisplatin nephrotoxicity. J. Nephropharmacol..

[15] Jones M.M., Basinger M.A., Holscher M.A. (1992). Control of the nephrotoxicity of cisplatin by clinically used sulfur-containing compounds. Fund. Appl. Toxicol..

[16] Lee S., Moon S.O., Kim W., Sung M.J., Kim D.H., Kang K.P., Jang Y.B., Lee J.E., Jang K.Y., Lee S.Y. (2006). Protective role of L-2-oxothiazolidine-4-carboxylic acid in cisplatin-induced renal injury. Nephrol. Dial. Transplant..

[17] Lu Y., Kawashima A., Horii I., Zhong L. (2005). Cisplatin-induced cytotoxicity in BSO-exposed renal proximal tubular epithelial cells: Sex, age, and species. Ren. Fail..

[18] Muldoon L.L., Walker-Rosenfeld S.L., Hale C., Purcell S.E., Bennett L.C., Neuwelt E.A. (2001). Rescue from enhanced alkylator-induced cell death with low molecular weight sulfur-containing chemoprotectants. J. Pharmacol. Exp. Ther..

[19] Park H.-M., Cho J.-M., Lee H.-R., Shim G.-s., Kwak M.-K. (2008). Renal protection by 3H-1,2-dithiole-3-thione against cisplatin through the Nrf2-antioxidant pathway. Biochem. Pharmacol..

[20] Quintanilha J.C.F., de Sousa V.M., Visacri M.B., Amaral L.S., Santos R.M.M., Zambrano T., Salazar L.A., Moriel P. (2017). Involvement of cytochrome P450 in cisplatin treatment: Implications for toxicity. Cancer Chemother. Pharmacol..

[21] Salama S.A., Abd-Allah G.M., Mohamadin A.M., Elshafey M.M., Gad H.S. (2021). Ergothioneine mitigates cisplatin-evoked nephrotoxicity via targeting Nrf2, NF-κB, and apoptotic signaling and inhibiting γ-glutamyl transpeptidase. Life Sci..

[22] Spitz D.R., Phillips J.W., Adamas D.T., Sherman C.M., Deen D.F., Li G.C. (1993). Cellular resistance to oxidative stress is accompanied by resistance to cisplatin: The significance of increased catalase activity and total glutathione in hydrogen peroxide-resistant fibroblasts. J. Cell. Physiol..

[23] Wu Y.J., Muldoon L.L., Neuwelt E.A. (2005). The chemoprotective agent N-acetylcysteine blocks cisplatin-induced apoptosis through caspase signaling pathway. J. Pharmacol. Exp. Ther..

[24] Zhang J.G., Zhong L.F., Zhang M., Ma X.L., Xia Y.X., Lindup W.E. (1994). Amelioration of cisplatin toxicity in rat renal cortical slices by dithiothreitol in vitro. Hum. Exp. Toxicol..

[25] Mandic A., Hansson J., Linder S., Shoshan M.C. (2003). Cisplatin induces endoplasmic reticulum stress and nucleus-independent apoptotic signaling. J. Biol. Chem..

[26] Yan M., Shu S., Guo C., Tang C., Dong Z. (2018). Endoplasmic reticulum stress in ischemic and nephrotoxic acute kidney injury. Ann. Med..

[27] Hsing C.-H., Tsai C.-C., Chen C.-L., Lin Y.-H., Po-Chun Tseng P.-C., Satria R.D., Lin C.-F. (2021). Pharmacologically inhibiting glycogen synthase kinase-3 ameliorates renal inflammation and nephrotoxicity in an animal model of cisplatin-induced acute kidney injury. Biomedicines.

[28] Perazella M.A. (2019). Drug-induced acute kidney injury: Diverse mechanisms of tubular injury. Curr. Opin. Crit. Care.

[29] Brady H.R., Kone B.C., Stromski M.E., Zeidel M.L., Giebisch G., Gullans S.R. (1990). Mitochondrial injury: An early event in cisplatin toxicity to renal proximal tubules. Am. J. Physiol..

[30] Guan J., Tong X., Zhang Y., Xu F., Zhang Y., Liang X., Jin J., Jing H., Guo L., Ni X. (2021). Nephrotoxicity induced by cisplatin is primarily due to the activation of the 5-hydroxytryptamine degradation system in proximal renal tubules. Chem.-Biol. Interact..

[31] Jiang M., Wang C., Huang S., Yang T., Dong Z. (2009). Cisplatin-induced apoptosis in p53-defificient renal cells via the intrinsic mitochondrial pathway. Am. J. Physiol..

[32] Kim D.H., Jung Y.J., Lee J.E., Lee A.S., Kang K.P., Lee S., Park S.K., Han M.K., Lee S.Y., Ramkumar K.M. (2011). SIRT1 activation by resveratrol ameliorates cisplatin-induced renal injury through deacetylation of p53. Am. J. Physiol..

[33] Kruidering M., van de Water B., de Heer E., Mulder G.J., Nagelkerke J.F. (1997). Cisplatin-induced nephrotoxicity in porcine proximal tubular cells: Mitochondrial dysfunction by inhibition of complexes I to IV of the respiratory chain. J. Pharmacol. Exp. Ther..

[34] Mukhopadhyay P., Horváth B., Zsengellér Z., Zielonka J., Tanchian G., Holovac E., Kechrid M., Vivek Patel V., Stillman I.E., Parikh S.M. (2012). Mitochondrial-targeted antioxidants represent a promising approach for prevention of cisplatin-induced nephropathy. Free Radic. Biol. Med..

[35] Park M.S., De Leon M., Devarajan P. (2002). Cisplatin induces apoptosis in LLC-PK1 cells via activation of mitochondrial pathways. J. Am. Soc. Nephrol..

[36] Qian W., Nishikawa M., Haque A.M., Hirose M., Mashimo M., Sata E., Inoue M. (2005). Mitochondrial density determines the cellular sensitivity to cisplatin-induced cell death. Am. J. Physiol..

[37] Tanabe K., Tamura Y., Lanaspa M.A., Miyazaki M., Suzuki N., Sato W., Maeshima Y., Schreiner G.F., Villarreal F.J., Johnson R.J. (2012). Epicatechin limits renal injury by mitochondrial protection in cisplatin nephropathy. Am. J. Physiol. Renal Physiol..

[38] Yang Y., Liu H., Liu F., Dong Z. (2014). Mitochondrial dysregulation and protection in cisplatin nephrotoxicity. Arch. Toxicol..

[39] Zhang J.-G., Lindup W.E. (1993). Role of mitochondria in cisplatin-induced oxidative damage exhibited by rat renal cortical slices. Biochem. Pharmacol..

[40] Zhang J.-G., Lindup W.E. (1994). Cisplatin nephrotoxicity: Decreases in mitochondrial protein sulfhydryl concentration and calcium uptake by mitochondria from rat renal cortical slices. Biochem. Pharmacol..

[41] Barry M.A., Behnke C.A., Eastman A. (1990). Activation of programmed cell death (apoptosis) by cisplatin, other anticancer drugs, toxins and hyperthermia. Biochem. Pharmacol..

[42] Bolisetty S., Traylor A.M., Kim J., Joseph R., Ricart K., Landar A., Agarwal A. (2010). Heme oxygenase-1 inhibits renal tubular macroautophagy in acute kidney injury. J. Am. Soc. Nephrol..

[43] Cummings B.S., Schnellmann R.G. (2002). Cisplatin-induced renal cell apoptosis: Caspase 3-dependent and -independent pathways. J. Pharmacol. Exp. Ther..

[44] Cummings B.S., McHowat J., Schnellmann R.G. (2004). Role of an Endoplasmic reticulum Ca^2+^-independent phospholipase A_2_ in cisplatin-Induced renal cell apoptosis. J. Pharmacol. Exp. Ther..

[45] Dong G., Luo J., Kumar V., Dong Z. (2010). Inhibitors of histone deacetylases suppress cisplatin-induced p53 activation and apoptosis in renal tubular cells. Am. J. Physiol..

[46] Wu W., Fu Y., Liu Z., Shu S., Wang Y., Tang C., Cai J., Dong Z. (2021). NAM protects against cisplatin-induced acute kidney injury by suppressing the PARP1/p53 pathway. Toxicol. Appl. Pharmacol..

[47] Li X.-M., Metzger G., Filipski E., Boughattas N., Lemaigre G., Hecquet B., Filipski J., Levi F. (1997). Pharmacologic modulation of reduced glutathione circadian rhythyms with buthionine sulfoximine: Relationship with cisplatin toxicity in mice. Toxicol. Appl. Pharmacol..

[48] Townsend D.M., Marto J.A., Deng M., Macdonald T.J., Hanigan M.H. (2003). High pressure liquid chromatography and mass spectrometry characterization of the nephrotoxic biotransformation products of cisplatin. Drug Metab. Dispos..

[49] Townsend D.M., Deng M., Zhang L., Lapus M.G., Hanigan M.H. (2003). Metabolism of cisplatin to a nephrotoxin in proximal tubule cells. J. Am. Soc. Nephrol..

[50] Hanigan M.H., Gallagher B.C., Taylor P.T., Large M.K. (1994). Inhibition of g-glutamyl transpeptidase activity by acivicin in vivo protects the kidney from cisplatin-induced toxicity. Cancer Res..

[51] Hanigan M.H., Gallagher B.C., Taylor P.T. (1996). Cisplatin nephrotoxicity: Inhibition of g-glutamyl transpeptidase blocks the nephrotoxicity of cisplatin without reducing platinum concentrations in the kidney. Am. J. Obstet. Gynecol..

[52] Townsend D.M., Hanigan M.H. (2002). Inhibition of g-glutamyl transpeptidase or cysteine conjugate b-lyase activity blocks the nephrotoxicity of cisplatin. J. Pharmacol. Exp. Ther..

[53] Zhang L., Hanigan M.H. (2003). Role of cysteine *S*-conjugate b-lyase in the metabolism of cisplatin. J. Pharmacol. Exp. Ther..

[54] Rooseboom M., Schaaf G., Commandeur J.N.M., Vermeulen N.P.E., Fink-Gremmels J. (2002). b-Lyase-dependent attenuation of cisplatin-mediated toxicity by selenocysteine Se-conjugates in renal tubular cell lines. J. Pharmacol. Exp. Ther..

[55] Cichocki J.A., Guyton K.Z., Guha N., Chiu W.A., Rusyn I., Lash L.H. (2016). Target organ metabolism, toxicity, and mechanisms of trichloroethylene and perchloroethylene: Key similarities, differences, and data gaps. J. Pharmacol. Exp. Ther..

[56] Wainford R.D., Weaver R.J., Stewart K.N., Brown P., Hawksworth G.M. (2008). Cisplatin nephrotoxicity is mediated by gamma glutamyltranspeptidase, not via a C-S lyase governed biotransformation pathway. Toxicology.

[57] Lash L.H. (2006). Mitochondrial glutathione transport: Physiological, pathological and toxicological implications. Chem.-Biol. Interact..

[58] Lash L.H., Putt D.A., Matherly L.H. (2002). Protection of NRK-52E cells, a rat renal proximal tubular cell line, from chemical-induced apoptosis by overexpression of a mitochondrial glutathione transporter. J. Pharmacol. Exp. Ther..

[59] Xu F., Putt D.A., Matherly L.H., Lash L.H. (2006). Modulation of expression of rat mitochondrial 2-oxoglutarate carrier in NRK-52E cells alters mitochondrial transport and accumulation of glutathione and susceptibility to chemically induced apoptosis. J. Pharmacol. Exp. Ther..

[60] Lash L.H., Putt D.A., Hueni S.E., Cao W., Xu F., Kulidjian S.J., Horwitz J.P. (2002). Cellular energetics and glutathione status in NRK-52E cells: Toxicological implications. Biochem. Pharmacol..

[61] Benipal B., Lash L.H. (2013). Modulation of mitochondrial glutathione status and cellular energetics in primary cultures of proximal tubular cells from remnant kidney of uninephrectomized rats. Biochem. Pharmacol..

[62] Panteix G., Beaujard A., Garbit F., Chaduiron-Faye C., Guillaumont M., Gilly F., Baltassat P., Bressolle F. (2002). Population pharmacokinetics of cisplatin in patients with advanced ovarian cancer during intraperitoneal hyperthermia chemotherapy. Anticancer Res..

[63] Urien S., Lokiec F. (2004). Population pharmacokinetics of total and unbound plasma cisplatin in adult patients. Br. J. Clin. Pharmacol..

[64] de Jongh F.E., Gallo J.M., Shen M., Verweij J., Sparreboom A. (2004). Population pharmacokinetics of cisplatin in adult cancer patients. Cancer Chemother. Pharmacol..

[65] Rajkumar P., Mathew B.S., Das S., Isaiah R., John S., Prabha R., Fleming D.H. (2016). Cisplatin concentrations in long and short duration infusion: Implications for the optimal time of radiation delivery. J. Clin. Diag. Res..

[66] Lash L.H., Lee C.A., Wilker C., Shah V. (2018). Transporter-dependent cytotoxicity of antiviral drugs in primary cultures of human proximal tubular cells. Toxicology.

[67] Lu S.C. (2009). Regulation of glutathione synthesis. Mol. Asp. Med..

[68] Devarajan N., Manjunathan R., Ganesan S.K. (2021). Tumor hypoxia: The major culprit behind cisplatin resistance in cancer patients. Crit. Rev. Oncol./Hematol..

[69] Fariss M.W., Reed D.J. (1987). High-performance liquid chromatography of thiols and disulfides: Dinitrophenyl derivatives. Methods Enzymol..

[70] Visarius T.M., Putt D.A., Schare J.M., Pegouske D.M., Lash L.H. (1996). Pathways of glutathione metabolism and transport in isolated proximal tubular cells from rat kidney. Biochem. Pharmacol..

